# On Functional Disorders of the Large Bowels and on Diseases Which They Induce

**Published:** 1830-01-01

**Authors:** 


### ON FUNCTIONAL DISORDERS OF THE LARGE BOWELS, AND ON CERTAIN DISEASES WHICH THEY OFTEN INDUCE. By *James Annesley*, Esq.

[ART. V.]

IN the 20th Number of this Journal, we brought the first volume of Mr. Annesley's work to a close, in four analytical articles which we dedicated to that stupendous publication. We now proceed to the second volume, the principal contents of which we shall lay before our readers, though not exactly in the order adopted by the author. Before entering on organic changes in the intestinal canal and some of the collatitious viscera, we shall first treat of certain functional disorders of the large bowels. The functional disorders here treated of, says Mr. A. "are chiefly characterised by a deficient tone or action—by a torpid state of the functions usually performed by the different tissues composing these viscera."

These disorders, he further observes, though little calculated to excite alarm at first, yet often lead to serious consequences in the end. The first subject of investigation is—

### I. ACCUMULATIONS OF MORBID SECRETIONS AND FÆCAL MATTERS IN THE LARGE BOWELS.

Even in health a remora of the secretions and excretions takes place in the cæcum and colon—and the construction of these parts proves that such remora was designed by the Great Artchitect of our frame. But unfortunately this remora too frequently goes to a morbid extent, producing not only mechanical but vital mischief. Our author thinks that among the immediate consequences of this torpid state of the bowels, is the retention of the mucous secretions poured out from their follicular glands, and the accumulation of mucus in the glands themselves subsequently, and in the ducts leading from them:—"hence the mucous follicles frequently become obstructed, distended, and subsequently inflamed and ulcerated." When, in addition to this accumulation of viscid secretions, the fæcal matters are also retained, still greater mischief ensues.

"The more fluid portions of the fæces, and of the secretions themselves, are then absorbed into the circulation, and carried either into the general current of blood, or, in the first instance, into that portion which flows into the portal vein, and which circulates through the liver. The consequences of this absorption of matters which are excrementitious and hurtful to the system may be readily inferred; and the effects it is calculated to produce upon the functions of the liver, and indeed upon the system generally, must be evidently injurious." 48.

Retention of the biliary secretion, Mr. A. observes, causes *icterus* in the slighter shades—a symptom not uncommon in tropical climates, where the liver pours out a large quantity of bile that is resorbed from the intestines into the general circulation.

"When accumulations, either of mucous secretions, or of the fæcal and other materials, or of all these combined, form in the cæcum and large intestines, the mucous follicles become obstructed, enlarged, and disposed to disease of a serious nature; the mucous tunic is impeded in the performance of its various functions; the muscular coats of the bowels become flaccid, and their irritability diminished; the accumulated materials enter into new combinations, give rise to gaseous productions, and at last degenerate into noxious matters; and thus the cæcum and cells of the colon are enormously distended by these materials, many of which have been collecting from a remote period, and by the flatus evolved from their decay and the new states of combination they are disposed to assume, from the presence of moisture, combined with a high temperature.

"The distension which frequently takes place in the colon and cæcum from these causes is often very great, and of itself productive of serious disorder. Nor is this surprising, when we consider the various connexions which the cæcum and colon have with the other abdominal viscera, and the manner in which the functions of these viscera may be even mechanically impeded by great distension of these bowels. When in this unnatural state, the cæcum and sigmoid flexure of the colon press upon the femoral nerves, and blood-vessels, the vena cava, and internal iliac veins, producing numbness, cramps, pains in the lower extremities, and even œdema, owing to the impeded return of blood through the veins. The ascending and descending portions of the loaded and distended colon press injuriously upon the kidneys and adjoining vessels, and occasion a dull aching and sense of weight in the loins, with disorder of the urinary secretion. When distension of the right flexure and transverse arch of the colon is present, the functions of the liver, the discharge of bile into the duodenum, and the states of the gall-bladder, the duodenum, and stomach, are very materially interfered with. If the accumulations in the arch and flexures of the colon be carried to their utmost, the healthy conditions of the stomach, duodenum, liver, gall-bladder, and biliary ducts, become very seriously deranged, the descent of the diaphragm is much impeded, and the disorder extended to the functions of the lungs and heart. Owing to this latter effect, together with the mechanical influence of the original cause upon the abdominal circulation, the return of blood from the head is retarded; and, as one of its most remote consequences, congestions on the brain, and effusions of serum from its membranes supervene." 49.

Mr. A. has no doubt that enlargements and other diseases of the mesenteric glands often originate in this manner—and are cured by appropriate means. When unhealthy chyle is thus formed, and excrementitious matters carried into the circulation, nutrition fails—the countenance becomes pale and afterwards sallow—the body wastes—and various disorders of the respiratory, digestive, and nervous systems ensue, leading the practitioner to suspect organic diseases, when only functional disorder obtains. The irritation produced by these accumulations sometimes induces diarrhœa—sometimes dysentery, "frequently terminating in ulceration of the bowel." Intestinal worms, and cutaneous complaints are often produced, Mr. A. thinks, by these accumulations, as also hypochondriacal and melancholic affections.

"In respect of the symptoms *indicating a loaded state of the cœcum and colon*, it is necessary that the practitioner should be well informed. We need not acquaint the experienced observer of disease that these symptoms are very various in different cases, and that the disorder of organs remote from the seat of disease will often be the chief cause which we may have of suspecting the nature of the original derangement. In all cases, an accurate examination should be made of the abdomen of the patient, commencing with the seat of the cæcum in the right iliac region, following the direction of the colon between the top of the ilium and right ribs, across the epigastric region, and under both hypochondria to the left side and left iliac fossa. If, in the course of our examination, pain be complained of, chronic inflammation should be suspected, and its existence judged of according to the symptoms present. If there be fulness evidently existing in the course now pointed out, or in the abdomen generally, and particularly if the fulness give a doughy sensation to the hand of the examiner, we may consider that the internal surface of the bowels is lined with sordes and accumulated secretions. If more or less hardness be perceived about the seat of the cæcum, or in any part of the course of the colon or its sigmoid flexure, then the accumulation of hardened fæces should be dreaded. But even in cases where the most careful examination furnishes no proofs of the existence of morbid matters in the prima via, we are not on that account to infer that they do not actually exist. Flatulence, either of the small or large intestines, frequently prevents the examination from being so successful as it would otherwise be; and even although the internal surface of the bowels may be much loaded, yet their calibre may also be so much contracted, or at least so little distended, as to give rise to little or no sensible fulness of the abdominal regions. Besides, it often requires very considerable tact to discover this species of derangement by manual examination —a tact which can be acquired only by experience." 54.

A long continuance of these accumulations generally occasions additional symptoms, as uneasiness, sense of weight, and distention of the abdomen; together with loss of appetite, inactivity, dull pain of the loins, weakness of the lower extremities, furred tongue, drowsiness, &c.

Daily evacuations from the bowels afford no proof that the bowels are daily evacuated. Fæcal matters will lurk in the cells and flexures of the colon for weeks, months, and probably for years. Although the bowels may be daily acted on. The irritation produced by these pent up matters often leads to frequent calls to stool, by which both patient and practitioner are deceived.

"Upon inspecting the stools in these cases they are generally more or less fluid, or are of a soft consistence, and apparently composed of hardened fæces, broken down amid a dark-coloured fluid. Sometimes they are nearly of a natural colour, but often brown, greenish-brown, or muddy; they are generally offensive. If in this state a gentle aperient be given, the stools are frequently to appearance not much disordered,—a circumstance which often misleads both the physician and the patient, and the disorder is therefore imputed to some other cause. If, instead of a gentle purgative, an active cathartic remedy be exhibited, the patient has frequent irritating calls to stool; the motions are watery, and loaded with a gelatinous mucus; and he often complains of tenesmus;—consequences which, equally with the former, tend to mislead. In these cases, the cause of disorder is frequently overlooked, and the employment of suitable medicines either not persisted in sufficiently long to be productive of advantage, or not at all resorted to.

"In cases of the description now under consideration, much discrimination is requisite in the choice of the kind of purgative which should be prescribed. If aperients and laxatives only be employed, they are seldom sufficiently powerful to remove the accumulated matters, and frequently they do little more than procure the discharge of the more watery parts of the fæcal contents, or the excrementitious materials more recently formed. If active cathartics be prescribed, they often occasion distress, by irritating the mucous surface so far as to excite considerable action of the muscular tunics of the bowels, and to occasion a firmer retentien of the accumulated matters in the cells of the colon, so that the more fluid portions of the fæces only are brought away, with the exhaled fluids and the mucous secretion which the irritating effects of the cathartic had very greatly increased." 57.

The author next adverts to the pathological condition of the bowels on which these accumulations depend. A torpid or inactive state of these viscera is connected, he observes, with debility of the frame generally, or of the bowels particularly. To this succeeds a defective secretion from the mucous membrane itself—and ultimately chemical decomposition of the pent-up matters.

"During the employment of purgatives in cases of this description, and the persisting in the use of them for a sufficient time, it is almost surprising to observe the quantity of viscid, tenacious mucus which is brought away along with fæcal matters which have evidently been long pent up in the cells of the colon. Sometimes the stools have a gelatinous appearance and consistence, from the quantity of this kind of mucus with which they abound. At other times this substance forms only a part of the stool, the rest consisting of fæcal, offensive matters, and a watery fluid, with broken down fæces: when such evacuations are observed, the mucus is often very ropy or glairy, particularly tenacious, and always deposited at the bottom of the vessel, owing apparently to its greater specific gravity. In such instances, a stick is required to ascertain its existence, when it may be raised along the sides of the vessel by the point of the stick in one or more tenacious, glairy masses. As respects colour, these mucous evacuations vary very remarkably. Sometimes they are of a deep green, passing into a greenish black: at other times they are of a yellowish green, and of every shade to a bright orange and pale yellow." 60.

Mr. Annesley in this place hints that the gelatinous and glairy state of the evacuations has been attributed by some of his colleagues to the irritation of the purgative medicines employed—and thus the purgative plan has been impugned. But, says he, "if the purgatives occasioned the state of the evacuations which we have now described, the continued employment of them must invariably increase the quantity of mucus excreted, instead of diminishing it, and thus augment the disorder, instead of removing it." We think this inference is a very strained one. Does a disease never cease under improper treatment? The irritability of a membrane may become exhausted by repeated application of irritants, and especially of purgatives, and thus the disease may appear to be cured by means that were by no means indicated. We do not say that the purgatives were improper in the cases under consideration; but we certainly do demur to the general conclusion which our author has drawn in this place.

In the following remarks we are happy to agree with Mr. Annesley.

"Not only has this particular mucous or gelatinous state of the stools been ascribed entirely to the purgatives used, but the greenish hue of the evacuations has also been imputed to the same cause ; namely, to the influence of calomel, when that particular purgative has been prescribed. That calomel actually has the effect of giving a greenish tinge to the alvine evacuations, we will not deny ; but we do contend, from an experience of this remedy as extensive as has ever been enjoyed by any single practitioner, that, when it gives a greenish tinge,—whether of a very dark or of a very light hue, or of any intermediate tint,—to the alvine evacuations, the secretions poured into the alimentary canal are of a morbid condition, requiring purgatives to carry them out of the system, and mercurial alteratives, or medicines operating in a similar manner, to restore the secretions to a healthy state.

"When mercurial preparations, especially calomel, mix with the morbid secretions lining the alimentary canal, and with the biliary and pancreatic juices, and more particularly if the bile have been detained for some time in the gall-bladder, or have otherwise acquired greater consistence, a deeper colour, and more acrid properties, a greenish tint of the evacuations is generally remarked, the deepness and darkness of the colour depending upon the quantity of bile, and the condition of the secretions of the bowels and of the functions of these viscera generally : but this condition is less to be imputed to the particular kind of medicine prescribed, than to the morbid condition of the matters collected in the bowels on which it acts. That such is the case, is proved by the circumstance of the stools assuming a healthy character after this particular purgative has been employed sufficiently long to carry off the morbid secretions and accumulations existing in the *primæ viæ*, and to correct the disordered state of function whence these conditions proceed." 62.

TREATMENT. The *indications* are sufficiently obvious—namely, the exhibition of purgatives for the removal of accumulations, and the prevention of their re-accumulation by proper diet and medicines. In respect to the first indication, our author conceives that the generality of practitioners stop far short of the point to which purgation should be carried, being misled by the reports of the patients or the appearance of the stools. Whatever truth there may be in this remark, we agree with Mr. A. that the best purgatives are those which procure "a full, bulky, but not frequent evacuation of the bowels." Such remedies restore strength when it fails from the presence of fæcal matters, instead of lowering it still farther :—a consequence that often results from watery purgations.

"In many cases of long-neglected complaints of the digestive organs, the internal surface of the bowels, particularly of the cæcum and cells of the colon, become so thickly coated with a tenacious and thick secretion, giving rise to disorder of the *primæ viæ*, or of some remote organs, as to require the continued and energetic action of those purgatives more especially which procure full and bulky evacuations, before healthy condition of the system is restored. It is precisely in cases of this description that full doses of calomel, given at bed-time, operate so beneficially ; for this medicine produces its purgative effects, as we have already shewn, by dissolving the tenacious secretions, by promoting the biliary secretion, and by increasing the secretions of the mucous surface generally,—thus preparing the accumulated matters, and the bowels themselves, for the operation of the purgatives which may be subsequently prescribed." 67.

On this as on numerous other occasions, our author gives a preference to compound powder of jalap, the bitter aperient mixture, castor oil, decoct. aloes comp. compound aloetic pill, aided by calomel, when we want to promote the intestinal secretions as well as evacuate the bowels. Till the stools assume a healthy character, the purgative plan is to be strenuously pursued.

"Nor should he be misled by the appearance of healthy motions from the operation of the first doses of purgatives which he has prescribed; for he shall often find that, although the stools are at first apparently natural, yet the continued operation of these medicines will succeed in bringing away morbid matters long pent up in the cæcum and cells of the colon, having a very dark or marbled appearance and putty-like consistence. In such cases, the indication is clear, and the continued action of purgatives obviously requisite. But when the stools contain the glairy, gelatinous, and viscid mucus already referred to, much more doubt is apt to attach itself to the mind of the practitioner; and he is more prone to be diverted from his object by the supposition that the state of the stools is the consequence of the purgatives employed. The source of this appearance of the motions we have already attempted to explain in the foregoing section; and even when it does not proceed from that source, it is to be imputed to the presence of some other cause of irritation in the *primæ viæ* than the purgative prescribed." 68.

Mr. A. assures his readers that it is a most dangerous error in the practitioner to forego the use of calomel when the motions assume a greenish or spinage-like hue after the administration of that medicine, as we may be assured that the secretions of the intestinal canal, and even of the liver are in fault. Injections to facilitate the discharge of these morbid and poisonous secretions are very beneficial, as they prevent a great deal of irritation in the bowels and consequent disorder of the whole system.

But, as it sometimes, perhaps not unfrequently happens, that the retention of accumulated matters in the large bowels depends on spasmodic contractions of the lower portions of the colon and rectum, so it will be necessary, in such cases, to use the mildest purgatives combined with antispasmodics, as hyosciamus, compound galbanum pill, ammonia, æther, &c. The enemata should be of a similar kind. In many cases, also, bitters, or even tonics—and sometimes wine, are necessary, at the time that we are exhibiting purgatives, in consequence of the debility which generally as well as locally prevails.

A series of cases are given by Mr. Annesley, in illustration of the observations in the text. Of these we shall only be able to insert one—and that the first on the list.

"COLONEL M——— had been some years at the Cape of Good Hope, and enjoyed tolerably good health on his passage to Madras. He suffered much inconvenience from the want of those comforts in the ship which are essential, and indeed common. It may be presumed, therefore, that these privations and annoyances called into action some lurking disease in the constitution; for he was attacked, immediately on his arrival at Madras, in 1820, with an affection of the bowels, characterised by frequent inclinations, without the power to relieve himself; the motions were sometimes constipated, at other times loose, with a dull, heavy, deep-seated pain in the abdomen; foul tongue; great prostration of strength, and wasting of the flesh. The pulse was good, and the skin natural; spirits very much depressed, though he was naturally a very lively man, and he had no inclination for exertion; he says that for some time past he has felt the sensation of being what is commonly called bilious, and has taken some medicine, but he had never been under any regular treatment. On examining the abdomen, there was no pain on pressure being made on the hypochondriac region or under the ribs, but there was fulness at the epigastrium, and less elasticity over the whole abdomen than in health, particularly at the cæcum, where we observed some degree of tumefaction. The tongue, he says, has been foul for a long time, particularly on getting up in the morning; and the motions, although natural to appearance, have been irregular. From the fulness and want of elasticity in the abdomen, and particularly the accumulation in the cæcum, the nature of the disease appeared quite clear, and we immediately commenced upon a purgative plan of cure.—℞ Calom. gr. x.; extract, colocynth, gr. jv.; syrup. q. s. Ft. pilul. h. s. s. Mist. purg. ℥jv. mane sumend. These had very little effect, and what passed was not in any degree morbid. The following pills were given :—*i. e.* pilul. aloët. cum calom. et antim. tart. no. 1. three times a day; the calomel repeated at bed-time, and the purging draught in the morning. These acted, but not fully, until they had been continued for eight days, when they began to bring away much morbid matter, viscid and tenacious, of a brown colour. The same treatment was persisted in for six days longer, by which time the medicines began to act more regularly and decidedly. The pills were continued, and the bitter aperient mixture given night and morning. After these had been taken for three days longer, making altogether three weeks, the motions put on a very different appearance: they became viscid and gelatinous, of various colours, pale green, dark green, and of an orange colour, with some heavy, clay-like matter, which sunk to the bottom of the vessel. He had great pain in passing these motions, and particularly before the medicines acted: this induced him to believe that the remedies disagreed with him, and it was with some difficulty that we could persuade him to continue them. They were, however, continued without interruption for a fortnight longer, the patient passing the same kind of matter daily, varying, however, in colour, from jet-black to pale green, and from that to light yellow. The green stools had much the appearance of the scum formed upon stagnant water, and could be raised in the same manner by a stick. The purgatives were changed occasionally to calomel at bed-time and castor oil in the morning, but the same plan of treatment was regularly persevered in. The quantity of viscid, gelatinous, and tenacious matter that passed away was almost incredible. The symptoms at one time became so extremely distressing as even to occasion faintness whilst at stool; but the quantity of morbid matter which was discharged would have deterred any person who had not witnessed many similar cases from following up the purging plan, and might have been considered as the effect of the medicines. Purgatives, however, were regularly given, with the occasional variation above noticed, for ten more days, when the motions became more natural, though still viscid, and he passed them without pain; his spirits also improved, but he was exceedingly reduced in strength. The following pills were now prescribed, and the mixture continued.—℞ Pilul. aloët., hydrarg., et ipecac., nocte maneque. Repet. mist. amar. cum sennâ, ℥ij. nocte maneque. Sago, arrow-root, and wine, are allowed.

"In six days from the period at which the above were prescribed, the motions became quite natural in appearance and odour; and no more viscid, tenacious matter was discharged. The pills and the bitter aperient mixture were continued every night; his appetite improved; and from this time he recovered, but continued the medicine for another week, when one pill only was given, and the aperient every second or third day. The mineral acids and cold infusion of bark were now prescribed, and in a fortnight he was quite well, and is now in England and in good health." 75.

We shall pursue the various subjects of this second volume in succeeding articles.

